# Sexual Experience Induces the Expression of Gastrin-Releasing Peptide and Oxytocin Receptors in the Spinal Ejaculation Generator in Rats

**DOI:** 10.3390/ijms221910362

**Published:** 2021-09-26

**Authors:** Takumi Oti, Ryota Ueda, Ryoko Kumagai, Junta Nagafuchi, Takashi Ito, Tatsuya Sakamoto, Yasuhiko Kondo, Hirotaka Sakamoto

**Affiliations:** 1 Department of Biological Sciences, Faculty of Science, Kanagawa University, Hiratsuka 259-1293, Kanagawa, Japan; 2 Ushimado Marine Institute (UMI), Graduate School of Natural Science and Technology, Okayama University, Ushimado, Setouchi 701-4303, Okayama, Japan; pw1746ro@s.okayama-u.ac.jp (R.U.); pj5v7k6c@s.okayama-u.ac.jp (J.N.); ti1922@yamada-bee.com (T.I.); ryu@uml.okayama-u.ac.jp (T.S.); 3 Department of Animal Sciences, Teikyo University of Science, Uenohara 409-0193, Yamanashi, Japan; qup.kuma.po@gmail.com (R.K.); ykondo@ntu.ac.jp (Y.K.)

**Keywords:** sexual experience, lumbosacral spinal cord, spinal ejaculation generator, brain–spinal cord neural circuits, gastrin-releasing peptide, oxytocin, male sexual activity

## Abstract

Male sexual function in mammals is controlled by the brain neural circuits and the spinal cord centers located in the lamina X of the lumbar spinal cord (L3–L4). Recently, we reported that hypothalamic oxytocin neurons project to the lumbar spinal cord to activate the neurons located in the dorsal lamina X of the lumbar spinal cord (dXL) via oxytocin receptors, thereby facilitating male sexual activity. Sexual experiences can influence male sexual activity in rats. However, how this experience affects the brain–spinal cord neural circuits underlying male sexual activity remains unknown. Focusing on dXL neurons that are innervated by hypothalamic oxytocinergic neurons controlling male sexual function, we examined whether sexual experience affects such neural circuits. We found that >50% of dXL neurons were activated in the first ejaculation group and ~30% in the control and intromission groups in sexually naïve males. In contrast, in sexually experienced males, ~50% of dXL neurons were activated in both the intromission and ejaculation groups, compared to ~30% in the control group. Furthermore, sexual experience induced expressions of gastrin-releasing peptide and oxytocin receptors in the lumbar spinal cord. This is the first demonstration of the effects of sexual experience on molecular expressions in the neural circuits controlling male sexual activity in the spinal cord.

## 1. Introduction

Mammalian male sexual function is controlled not only by brain neural circuits but also by spinal cord centers, including the spinal ejaculation generator (SEG) located in the lamina X, and a portion of VII of the upper lumbar spinal cords (L3 and L4 levels) [1,2]. The primary component of SEG is a population of neurons called lumbar spinothalamic (LSt) neurons, which are believed to function to convey peripheral signals to the brain through direct projection from the lumbar spinal cord to the thalamus [1]. Specific toxin treatments that selectively destroy LSt neurons completely eliminate ejaculation in rats [1]. LSt neurons are much more prominent in male rats than in female rats and they are involved in the expression of galanin [1,2,3], cholecystokinin [4,5], enkephalin [6], and gastrin-releasing peptide (GRP) [7].

We reported in our previous study that neurons located in the dorsal lamina X of the lumbar spinal cord (dXL) overlapped with SEG project axons to the parasympathetic nervous system in the lower lumbar and upper sacral spinal cords (at the L5–L6 and S1 levels), the sacral autonomic nucleus (SAN), which controls male sexual functions such as erection and ejaculation in mice [8], rats [7,9], Suncus [10], and macaque monkeys [11]. Hypothalamic neurons that express nonapeptide oxytocin (OXT) have been reported to send axons not only to the brain region but also to the lower spinal cord—the SAN and the somatic spinal nucleus of the bulbocavernosus (SNB) that innervate the bulbocavernosus muscle attached to the base of the penis. It has already been suggested that these OXT projections are involved in penile function [12,13]. It is therefore considered plausible that the dXL and SNB systems, which are localized at similar levels in the lumbosacral spinal cord, have direct and/or indirect interaction with each other to modulate male sexual functions. The dXL neurons appear to generate ejaculation behavior by synchronizing both autonomic and somatic centers such as SAN and SNB in the lumbosacral spinal cord. Recently, we had reported that hypothalamic OXT neurons project to the upper lumbar spinal cord and activate lumbar neurons that express OXT receptors (OXTRs) and then facilitate male sexual functions such as erection and ejaculation [14].

Substantial evidence supports the claim that the first sexual experience significantly facilitates male sexual activity in rats [15]. Sexual experience is essential in indicating a positive preference for odors in estrous females [16]. The lesion of the medial amygdala in sexually naïve male rats severely impairs copulatory behavior, but not in sexually experienced males [17,18]. Sexual experience is also known to affect synaptic plasticity and spinogenesis in the medial preoptic area (mPOA) [19,20]. Furthermore, it has been reported that sexual experience increases the *Oxtr* expression in the mPOA and ventromedial hypothalamus of male rats [21]. These results suggest that sexual experience promotes male sexual activity through altering gene expression such as OXT and OXTR of the neural circuits that control male sexual activity in the hypothalamus.

In this study, we focused on dXL neurons that are innervated by hypothalamic OXT-ergic neurons controlling male sexual function and examined whether sexual experience affects such male-specific neural circuits in the spinal cord. Here, we demonstrated that sexual experience in male rats alters the expression levels of GRP and OXTR in dXL neurons.

## 2. Results

### 2.1. Experiment 1: Changes in the Activation of dXL Neurons between Naïve and Sexually Experienced Male Rats

**Figure 1:**
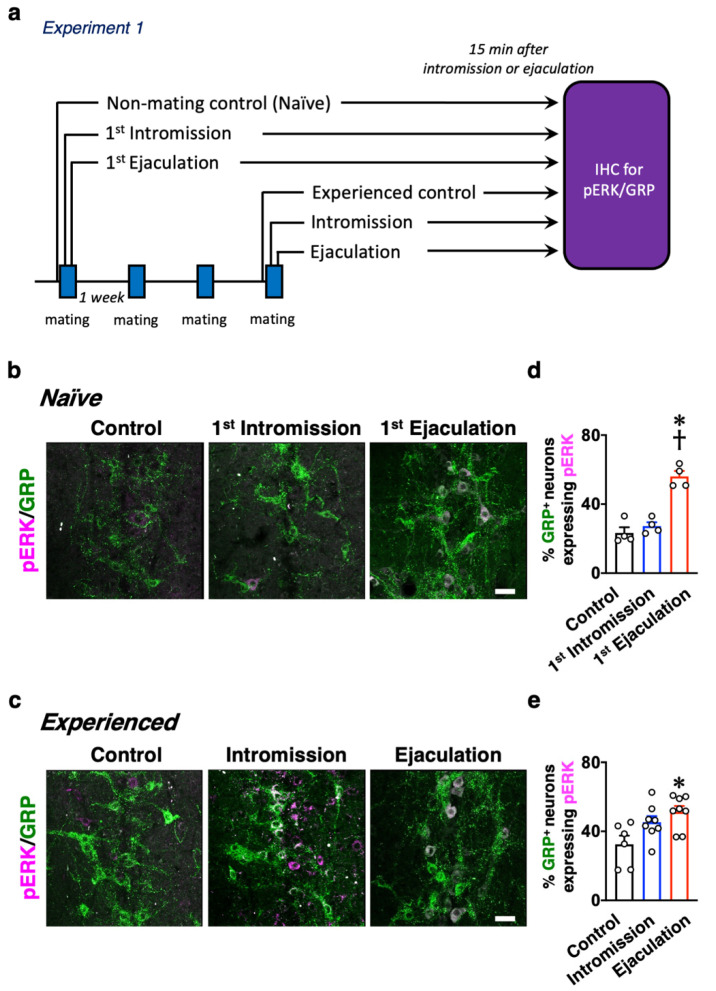
(**a**) Experimental paradigm of Experiment 1: changes in the activation of dXL neurons between naïve and sexually experienced male rats. Activation of spinal GRP-positive (^+^) neurons after sexual behavior in male rats with or without sexual experience. (**b**) Expression of phosphorylated extracellular signal-related kinases 1 and 2 (pERK) (magenta) in spinal GRP^+^ neurons (green) after sexual behavior of sexually naïve males. Left panel, Control. Middle panel, 1st-Intromission group. Right panel, after 1st Ejaculation. (**c**) Expression of pERK (magenta) in spinal GRP^+^ neurons (green) after sexual behavior of sexually experienced males. Left panel, Control. Middle panel, mounts and intromission only. Right panel, after ejaculation. (**d**) In naïve males, ejaculation significantly increased pERK expression in GRP^+^ neurons compared to that in the Control and Intromission groups (data presented as mean ± standard error of the mean (SEM) and individual dots; *n* = 4 per group). (**e**) In experienced males, Ejaculation significantly increased pERK expression in GRP^+^ neurons compared to that in the Control and Intromission groups (data presented as mean ± SEM and individual dots; *n* = 6 in Control group, *n* = 8 in the Intromission group, *n* = 8 in the Ejaculation group). No differences in any of the parameters were observed between the groups with or without sexual experience (two-way ANOVA, *F*_2,28_ = 4.10, * *p* < 0.05 vs. Control, † *p* < 0.05 vs. Intromission). Scale bar: 50 µm.

Focusing on sexual experience or the lack of it, some experiments were conducted, as indicated in Figure 1a, so as to analyze the activation of dXL neurons after sexual behavior in male rats. Male rats were immediately separated after their first mount with the penile insertion to the vagina (intromission) as an index of erection (the 1st-Intromission group), or the male rats were sacrificed immediately after the first ejaculation (the 1st-Ejaculation group). The male rats were perfusion fixed for 15 min after each behavior. The control rats were placed in the observation cage, and they were not presented with estrous females. Next, the rats that ejaculated two or more times in the three mating sessions were designated as experienced males (the sexually experienced group). The experienced male rats were separated immediately after 9 intromissions or the first ejaculation. The male rats were perfusion fixed for 15 min after each behavior. For the experienced male rats, a control group was also maintained, in which the estrous female rat was not presented in the observation cage as in the inexperienced group. Double immunofluorescence was performed for each spinal cord sample using antibodies against GRP, a marker for dXL neurons, and phosphorylated extracellular signal-related kinases 1 and 2 (pERK), which is a marker protein for neural activation (Figure 1b,c). The expression of pERK (magenta) in GRP-positive (^+^) neurons (green) was observed only in the first ejaculated rats (the 1st-Ejaculation group), and almost no activation of GRP^+^ neurons was recorded in the first intromission rats (the 1st-Intromission group) (Figure 1b,d; Table 1). In contrast, in the experienced male rats, GRP^+^ neurons were activated in both the Intromission and Ejaculation groups, and the ratio of activated GRP^+^ neurons (the number of pERK^+^ cells in the GRP^+^ cells) was significantly higher in both the Intromission and Ejaculation groups relative to those in the Control group (two-way analysis of variance (ANOVA), *F*_2,28_ = 4.10, * *p* < 0.05 vs. Control, † *p* < 0.05 vs. Intromission. Figure 1c,e; Table 1). For the experienced male rats, no statistical difference was noted in the ratio of activated GRP^+^ neurons in the Intromission and Ejaculation groups (Table 1).

### 2.2. Experiment 2: Effects of Sexual Experience on the dXL Neuron System

**Figure 2:**
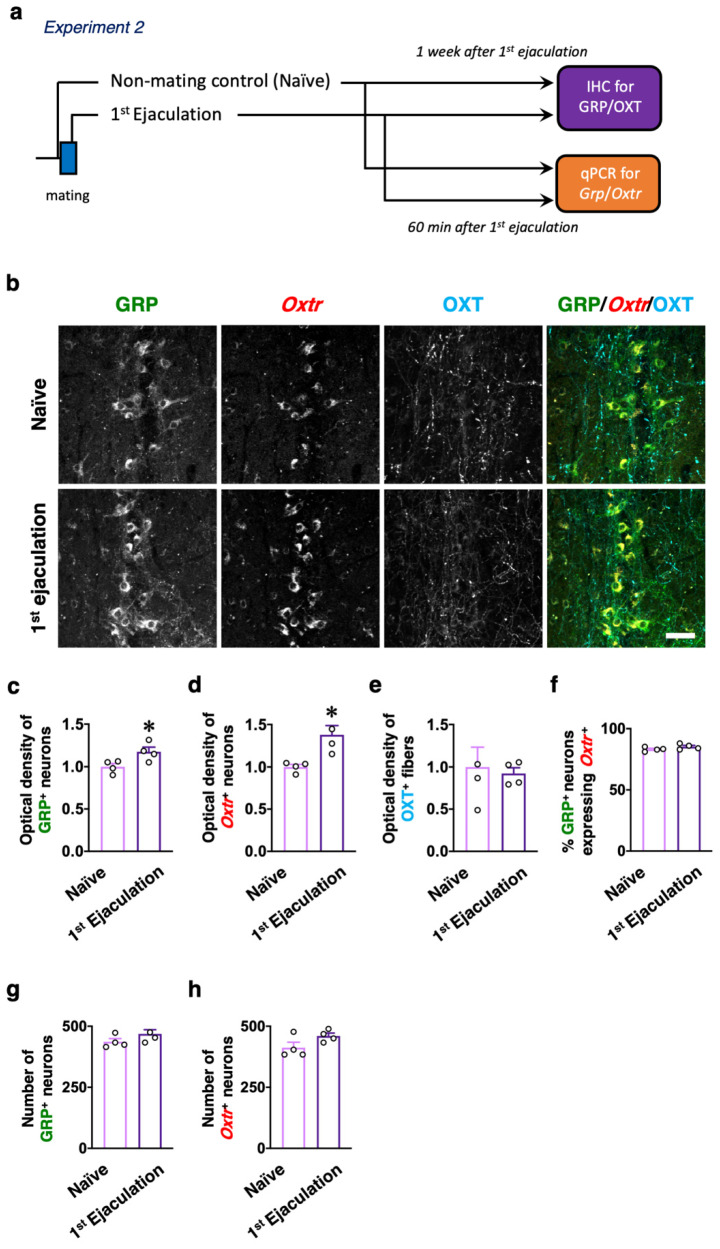
(**a**) Experimental paradigm of Experiment 2: effects of sexual experience on the dXL neuron system. (**b**) Immunohistochemistry for GRP and OXT in the upper lumbar spinal cord of *Oxtr*-YFP transgenic (Tg) male rats with or without sexual experience (*n* = 4, respectively). GRP-immunoreactivity (green) and YFP signals (*Oxtr*) (red) were higher in 1st-Ejaculation Tg male rats than in Naïve Tg male rats. No difference was noted in the intensity of OXT-positive (^+^) fibers (Cyan). In this study, we detected the immunofluorescence of OXT-neurophysin as a marker for OXT neurons. Scale bar: 100 µm. (**c**) The intensity of GRP^+^ neurons significantly increased in 1st-Ejaculation Tg male rats compared to Naïve Tg male rats (unpaired *t*-test, *t*_6_ = 2.65, * *p* < 0.05 vs. Naïve). (**d**) The intensity of *Oxtr*^+^ neurons significantly increased in 1st-Ejaculation Tg male rats compared to Naïve Tg male rats (unpaired *t*-test, *t*_6_ = 3.42, * *p* < 0.05 vs. Naïve). (**e**) No statistical difference was noted in the intensity of OXT^+^ fibers between 1st-Ejaculation and Naïve Tg male rats (unpaired *t*-test, *t*_6_ = 0.32). (**f**–**h**) No statistical difference was noted in the ratio of GRP^+^/ *Oxtr*^+^ (**f**), number of GRP^+^ neurons (**g**), or number of *Oxtr*^+^ neurons (**h**) between 1st-Ejaculation and Naïve Tg male rats (unpaired *t*-test, *t*_6_ = 1.51 in f, *t*_6_ = 1.51 in g, *t*_6_ = 1.90 in h).

**Figure 3:**
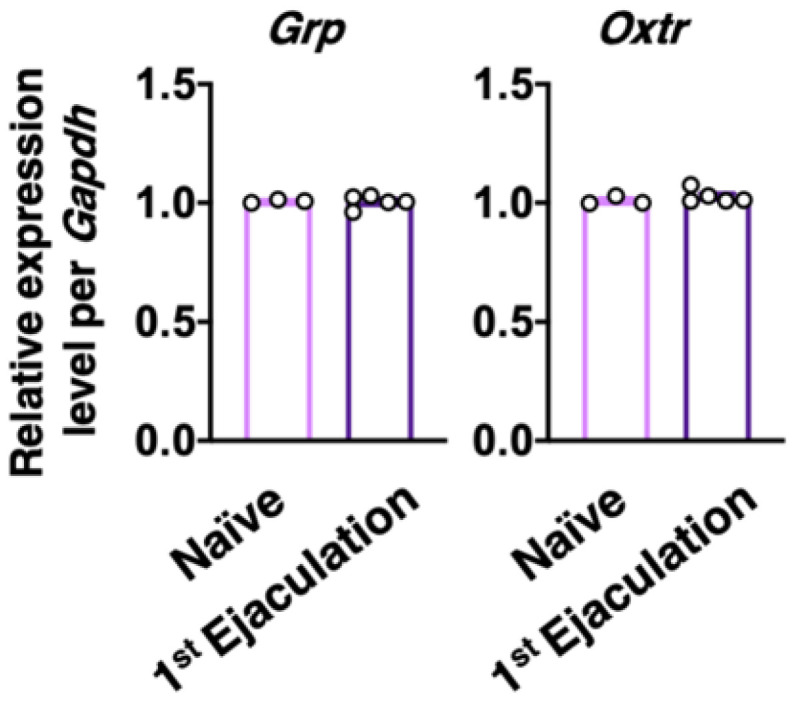
Effect of sexual experience on the *Grp* and *Oxtr* mRNA expression in the somal region of dXL neurons. No statistical difference was noted in the *Grp* and *Oxtr* mRNA expression between Naïve male rats (*n* = 3) and 1st-Ejaculation male rats (*n* = 5) (unpaired *t*-test, *t*_6_ = 0.15 in *Grp*, *t*_6_ = 0.93 in *Oxtr*).

**Figure 4:**
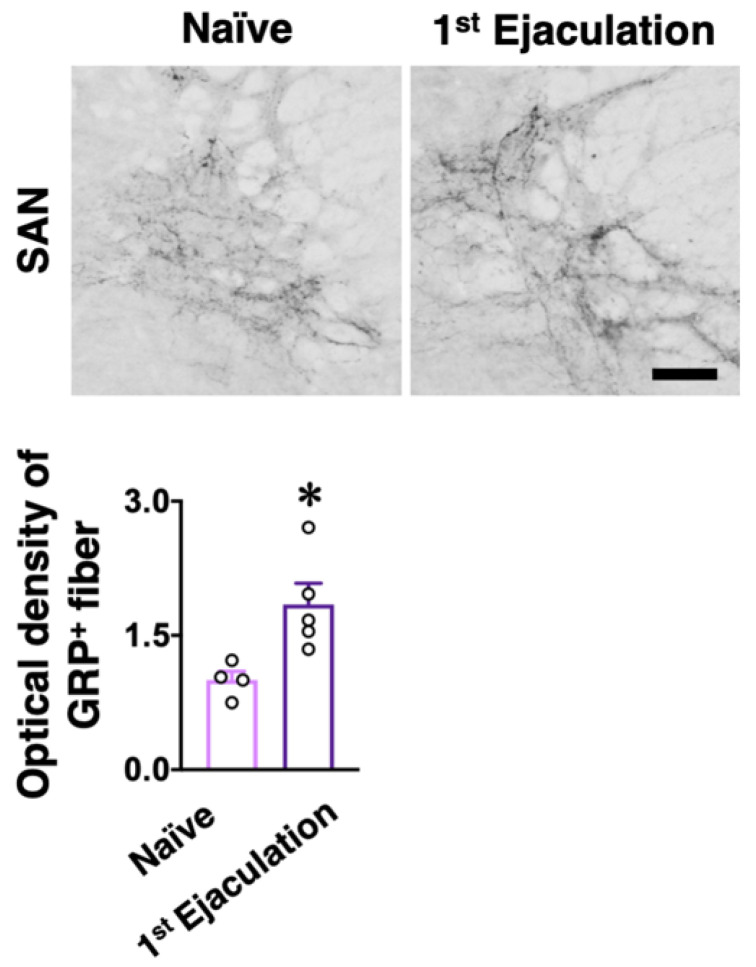
The effects of sexual experience on GRP-positive (^+^) fibers in the SAN. (**Upper panel**) The GRP^+^ fibers were higher in 1st-Ejaculation male rats (*n* = 5) than in Naïve male rats (*n* = 4). Scale bar: 50 µm. (**Lower panel**) Semi-quantitative analysis revealed that GRP^+^ fibers were significantly increased in 1st-Ejaculation male rats than in Naïve male rats (unpaired *t*-test, *t*_7_ = 3.00, * *p* < 0.05 vs. Naïve).

Male rats who ejaculated for the first time (the first ejaculation) during mating and gained sexual experience were fixed 1 week after their first ejaculation (1st-Ejaculation group) (Figure 2a). In the control group, naïve male rats were placed in the observation cage but they were not presented with any estrous females. We examined changes in several molecular expressions (i.e., GRP, OXTR, and OXT) activated by the first ejaculation (sexual experience) in dXL neurons. *Oxtr*-yellow fluorescent protein (YFP) transgenic (Tg) rats [14] were used to histologically analyze the expression of OXTR. GRP-immunoreactivity (green) was higher in the 1st-Ejaculation group than in the Naïve group (unpaired *t*-test, *t*_6_ = 2.65, * *p* < 0.05 vs. Naïve, Figure 2b,c). The expression of *Oxtr* (red) was also higher in the 1st-Ejaculation group than in the Naïve group (unpaired *t*-test, *t*_6_ = 3.42, * *p* < 0.05 vs. Naïve, Figure 2b,d). In contrast, no obvious differences were noted in OXT-immunoreactivity (Cyan) (unpaired *t*-test, *t*_6_ = 0.32, *p* = 0.76, Figure 2b,e). In addition, no obvious effects of sexual experience were noted on the expression of *Oxtr* in GRP^+^ neurons (unpaired *t*-test, *t*_6_ = 1.51, *p* = 0.18, Figure 2b,f). No effects of sexual experience were observed on either the number of GRP^+^ neurons or the *Oxtr*^+^ neurons between the Naïve group and the 1st-Ejaculation group (unpaired *t*-test, *t*_6_ = 1.51, *p* = 0.18 in f, *t*_6_ = 1.90, *p* = 0.11 in g, Figure 2g,h). Next, changes in *Grp* mRNA and *Oxtr* mRNA expression after sexual experience were analyzed through the real-time quantitative PCR (qPCR) technique, where the Naïve group was placed in the observation cage alone, and the 1st-Ejaculation group was separated immediately after the first ejaculation. The rats were sacrificed 60 min after ejaculation (Figure 2a). As a result, no obvious changes in the mRNA expressions of *Grp* and *Oxtr* were observed in the lumbar spinal cord (L3–L4 level; somal region of dXL neurons) (Figure 3). Furthermore, we analyzed GRP^+^ projections to the SAN involved in the parasympathetic function in the spinal cord. GRP-immunoreactivity in the SAN was found to be significantly higher in the 1st-Ejaculation group (unpaired *t*-test, *t*_7_ = 2.30, *p* < 0.05, Figure 4).

### 2.3. Experiment 3: Evaluation of Whether Reflexive Erection or Ejaculation Activates dXL Neurons

**Figure 5:**
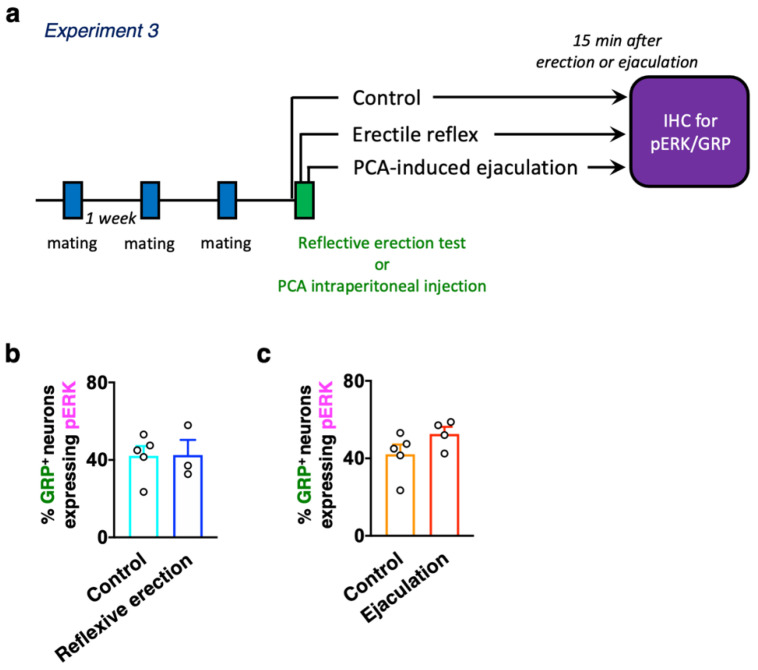
(**a**) Experimental paradigm of Experiment 3: evaluation of whether reflexive erection- or non-erection-induced ejaculation activates dXL neurons. (**b**) No statistical difference was noted in the ratio of activated spinal GRP-immunoreactive neurons between the Control (*n* = 5) and Reflexive erection groups (*n* = 3) (unpaired *t*-test, *t*_6_ = 0.05). (**c**) There was no statistical difference in the ratio of activated spinal GRP-immunoreactive neurons between the Control (*n* = 5) and Ejaculation groups (*n* = 4) (unpaired *t*-test, *t*_7_ = 1.60).

Finally, double immunofluorescence to determine the expressions of GRP and pERK was performed to evaluate the relationship between erection or ejaculation and the activation of dXL neurons. Male rats with three or more sexual behavior tests (with at least two ejaculations) were used in this study (Figure 5a). In the reflexive erection test, we investigated whether erections alone activated dXL neurons using male rats who were separated immediately after the third erection clusters. Male rats were perfusion fixed for 15 min after the last erection (Figure 5a). The control rats were gently restrained in the supine position with surgical tape, but their penises were not stimulated. Double immunofluorescence for GRP and pERK expression showed no significant effect of erections on the number of GRP^+^ neurons expressing pERK (unpaired *t*-test, *t*_6_ = 0.05, *p* = 0.96, Figure 5b and Table 2). We further evaluated the effect of ejaculation on the activation of dXL neurons by using a *p*-chloroamphetamine (PCA)-induced ejaculation model [22] with almost no erection (Figure 5a). Ejaculation was inducible through intraperitoneal administration of PCA [22]. Most of the anesthetized male rats showed an ejaculatory reflex of approximately 6 min after PCA administration [22]. No significant effect of ejaculation on the number of GRP^+^ neurons expressing pERK was also detected (unpaired *t*-test, *t*_7_ = 1.60, *p* = 0.15, Figure 5c and Table 2).

## 3. Discussion

**Figure 6:**
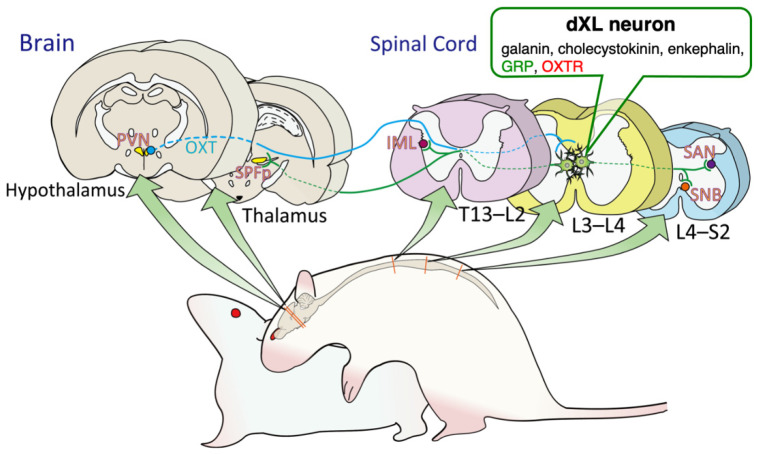
Schematic illustrations of the brain–spinal neural circuits that control male sexual activity in rats. Neurons located in the dorsal lamina X of the lumbar spinal cord (dXL) express galanin, cholecystokinin, enkephalin, gastrin-releasing peptide (GRP), and oxytocin receptor (OXTR). dXL neurons can also be identified as lumbar spinothalamic (LSt) neurons projecting from the lumbar spinal cord to the thalamus. In addition, dXL neurons project to both parasympathetic (sacral autonomic nucleus [SAN]) and sympathetic (intermediolateral cell column [IML]) systems in the thoracic spinal cord (the T13–L2 level) preganglionic neurons and sexual motor neurons (the spinal nucleus of the bulbocavernosus, SNB) located in the lower lumbar and upper sacral spinal cord (the L4–S2 level). In the thalamus, the parvocellular portion of the subparafascicular thalamic nucleus (SPFp) may receive sexual information from the afferent inputs of spinal dXL neurons.

As already reported, sexual experience facilitates male sexual activity in rats [15]. To elucidate the mode of action in the spinal cord in addition to that in the brain, we examined the effects of sexual experience on the dXL neuron system controlling penile function at the spinal cord level (Figure 6). We found that approximately two-fold more GRP neurons were activated in the ejaculation group than in the control and intromission groups in naïve males. In contrast, in male rats with sexual experience, activation of GRP neurons increased by 60% in the ejaculation group and by 40% in the intromission group as compared with that in the control group. An increase in Fos^+^ cell proportion after exposure to bedding soiled by an estrous female was recorded in the POA, the bed nucleus of the stria terminalis, and the nucleus accumbens in sexually experienced, but not in naïve, male rats [16]. Similarly, a different proportion of dXL neurons was activated depending on the level of sexual experience, suggesting that the spinal circuit for male sexual function is modulated by male sexual experiences such as intromission and ejaculation. dXL neurons have been known to control ejaculation via innervation to the SAN and spinal motor nuclei, which are involved in penile function [23]. They are also known to be regulated by efferent inputs from hypothalamic OXT [14]. Sexual experience may raise excitability in dXL neurons by these OXT-ergic efferents originating from the hypothalamus, consequently promoting male sexual activity through increased efficiency of innervation to the SAN and/or SNB. Alternatively, sexual experience may immediately switch the autonomic function from erection (parasympathetic) to ejaculation (sympathetic) by activating dXL neurons.

In the mPOA of male rats, sexual experience induces the expression of 20 or more genes but reduces the expression of more than 10 genes [24]. Furthermore, sexual experience increases the expression of *Oxtr* mRNA in the mPOA and the ventromedial hypothalamus in male rats [21]. In dXL neurons, it is possible that sexual experience can increase OXTR expression at the protein level, although changes in the *Oxtr* mRNA level could not be observed. Similarly, GRP expression levels were increased by sexual experience in dXL neurons. Sexual experience also appeared to affect spinal neural circuits involved in male sexual activity. In sexually experienced rats, dXL neurons were activated not only by ejaculation but also by intromissions. On the other hand, in naïve rats, dXL neurons were activated only by ejaculation. The activation of dXL neurons during the first ejaculation (or the first sexual experience) may imply the occurrence of changes in gene expression patterns. In addition to GRP, galanin, cholecystokinin, and enkephalin are expressed in dXL neurons [1,3,4,5,6,7]. Sexual experience may alter the expressions of these neuropeptides. Transcriptome analysis of dXL neurons could reveal the comprehensive neural mechanisms of the effect of the first sexual experience on the SEG system and the modification of male sexual activity.

Spinal cord injury (SCI) at the thoracic level does not influence the level of galanin expression in dXL neurons, but it reduces GRP expression [25]. Different regulatory mechanisms may hence be involved in the expression of galanin and GRP in dXL neurons [25,26]. SCI in the upper spinal cord (e.g., the thoracic spinal cord) possibly eliminates efferent innervation to dXL neurons from the brain. The expression of GRP in dXL neurons appears to be affected by brain function because SCI at the thoracic level significantly reduced GRP expression in dXL neurons [25]. It is already well known that the spinal transection at the lower thoracic level significantly increases the number of reflexive penile erections because the nerve connection between the brain and the lumbosacral spinal cord is damaged and the erectile-suppressing effect from the brain does not function [27]. Therefore, hyperactivity in penile function may induce increased GRP expression in dXL neurons.

To separately evaluate the involvement of dXL neurons in erection and ejaculation, we applied the reflexive erection test and PCA to induce ejaculation. The ratio was kept at approximately 50%, consistent with the ratio of activated GRP^+^ neurons after intromissions or after ejaculation in sexually experienced rats (Figure 1 and Figure 5; Table 1 and Table 2). In a subpopulation of dXL neurons, male sexual experience may lower the burst threshold and increase its sensitivity to activation at ejaculation. dXL neurons project axons not only to the parasympathetic nervous system of the lumbosacral spinal cord (erection), but also to the sympathetic nervous system of the thoracic spinal cord (ejaculation) [1,28]. dXL neurons may be functionally orchestrating/switching between the parasympathetic and sympathetic systems, and, when the excitement level reaches a threshold, they become increasingly activated by erections to generate ejaculation. Further attention needs to be paid to behavioral studies using galanin-Cre and/or GRP-Cre mice combined with the AAV-gene delivery system (e.g., optogenetics, pharmacogenetics, and/or fiber photometry). These results together demonstrate the activation of dXL neurons with a higher time resolution during a functional shift from erection (parasympathetic) to ejaculation (sympathetic).

## 4. Materials and Methods

All experiments were performed following the “Guiding Principles for Care and Use of Animals in the Field of Physiological Sciences” of the Physiological Society of Japan and were approved by the local Animal Experiment Committees of the Okayama University and the Teikyo University of Science. All efforts were made to minimize animal suffering and the number of animals used for the studies.

### 4.1. Animals

In experiment 1, adult male wild-type (Wt) rats of the Long–Evans strain (Institute for Animal Reproduction, Ibaraki, Japan) were used (Naïve: *n* = 4 per group and Experienced: *n* = 6 in Control group and *n* = 8 in each Intromission and Ejaculation groups). In experiment 2, adult male *Oxtr* promoter-human heparin-binding epidermal growth factor-like growth factor (human diphtheria toxin receptor; Dxtr)-ChR2-YFP BAC (*Oxtr*-YFP) Tg rats [14] (Wistar strain) bred in the animal facilities of Okayama University were used for IHC analysis of the upper lumbar spinal cord (*n* = 4 each in Naïve and 1st-Ejaculation groups). Adult male Wt rats of the Wistar strain (Charles River Japan, Yokohama, Japan) were used for qPCR analysis (*n* = 4 in Naïve group and *n* = 5 in 1st-Ejaculation group) and for IHC analysis of the lumbosacral spinal cord (*n* = 3 in Naïve group and *n* = 5 in 1st-Ejaculation group). All rats were maintained on a 12 h light/dark cycle and provided with unlimited access to water and rodent chow.

### 4.2. Effects of Sexual Experience on the Activation of dXL Neurons

To obtain male rats that ejaculated for the first time or male rats that demonstrated intromission for the first time, a few male rats were separated immediately after their first mount with the penile insertion to the vagina (intromission) as an index of erection (the 1st-Intromission group), or they were sacrificed immediately after their first ejaculation (the 1st-Ejaculation group). The male rats were perfusion fixed for 15 min after each behavior (Figure 1a). The controls were placed in the observation cage, but they were not presented with estrous females. Furthermore, the rats who ejaculated two or more times in three mating sessions were designated as experienced males (the sexually experienced group) (Figure 1a). Experienced male rats were separated immediately after nine intromissions or their first ejaculations. Male rats were perfusion fixed for 15 min after each behavior. For the inexperienced male rats, a control group was prepared in which the estrous female rats were not presented in the observation cage as in the inexperienced group.

### 4.3. Protein Expression of GRP, OXT, and YFP in the Lumbar Spinal Cord

Male rats that ejaculated for the first time (the first ejaculation) during mating and thereby gained sexual experience were fixed 1 week after their first ejaculation (1st-Ejaculation group) (Figure 2a). In the control group, naïve male rats were placed in the observation cage and they were not presented with estrous female rats.

### 4.4. Effects of Sexual Experience on the Grp and Oxtr mRNA Expressions in the Upper Lumbar Spinal Cord

Changes in *Grp* mRNA and *Oxtr* mRNA expression after male sexual experience were analyzed by the quantitative PCR (qPCR) technique with the Naïve group placed in the observation cage alone, while the 1st-Ejaculation group was separated immediately after the first ejaculation. The rats were sacrificed 60 min after ejaculation under deep anesthesia with 100 mg/kg body weight of an intraperitoneal sodium pentobarbital injection for real-time qPCR analyses. RNA extraction and real-time qPCR analysis were performed according to our established methods [29]. Lumbar spinal cords (L3–L4 level) were quickly removed and placed on an ice bath. The dissected tissues were immediately frozen using powdered dry ice. The preparations were mounted in a cryostat (CM3050 S; Leica, Nussloch, Germany) and the lamina X region was dissected along the rostrocaudal axis by using a stainless-steel needle (outer diameter, 1.2 mm; inner diameter, 0.94 mm). The dissected tissues were stored at −80 °C until RNA extraction. Total RNA was extracted using the Illustra RNAspin Mini RNA Isolation Kit (GE Health Care, Buckingham, UK) according to the manufacturer’s protocol. The concentration of total RNA was measured using the Qubit RNA Assay Kit (Thermo Fisher Scientific, Waltham, MA, USA). First-strand cDNA was synthesized from 250 ng of total RNA with random primers using the Omniscript RT Kit (Qiagen, Hilden, Germany). To assess *Grp, Oxtr,* and *Gapdh* expressions in the lumbar spinal cord of the Wt Wistar rats with or without sexual experience (Naïve; *n* = 3, Experienced; *n* = 5), a 15 µL reaction volume consisting of 1X TaqMan Universal PCR Master Mix (Applied Biosystems, Foster, CA, USA), 300 nM each of forward and reverse primers, and 200 nM of TaqMan probe was used. We used the TaqMan qPCR methodology for rat *Grp, Oxtr,* and *Gapdh* (gene expression assays no. Rn00592059, Rn005635037_m1, and Rn99999916; amplicon lengths 75, 60, and 87, respectively). Amplification was performed at 95 °C for 10 min and 40 cycles with 95 °C for 15 s and 60 °C for 1 min. The data of qPCR were analyzed using the CFX96 qPCR Detection System (Bio-Rad Laboratories, Hercules, CA, USA). The expression in each reaction was normalized by the expression of *Gapdh* as an internal control. Duplicate qPCR analysis was performed for each sample.

### 4.5. The Activation of dXL Neurons after Reflexive Erection or PCA-Induced Ejaculation

Male rats with three or more sexual training tests (with at least two ejaculations) were used in this study. In the reflexive erection test, we investigated whether erections alone could activate dXL neurons using male rats separated immediately after their third erection clusters. Male rats were perfusion fixed for 15 min after the last erection (Figure 5a). Control rats were gently restrained in the supine position by using surgical tape, but their penises were not stimulated. In addition, with reference to a previous study [22], PCA (C9635; Sigma, St. Louis, MO, USA; 20 mg/kg) was intraperitoneally administered to sexually experienced rats in order to induce ejaculation with almost no erection. The rats, 15 min after ejaculation, were quickly perfusion fixed for immunohistochemical analyses.

### 4.6. Sexual Behavior Test

For sexual behavior tests, stimulus female rats were ovariectomized, and estradiol benzoate (5 µg/0.1 mL sesame oil) was subcutaneously injected 2 days prior to testing. Progesterone (500 µg/0.1 mL sesame oil) was subcutaneously injected 4–6 h before testing so as to induce sexual receptivity. Sexual behavior tests were performed for 30 min, and the latency of the first mount, intromission, and ejaculation, as well as the number of mounts, intromissions, and ejaculations, were enumerated.

### 4.7. Immunohistochemistry and Immunofluorescence

The experimental rats were deeply anesthetized with intraperitoneal injections of sodium pentobarbital (50 mg kg^−h^ body weight) and perfused via the left ventricle with 100 mL of physiological saline, followed by 200 mL of 4% formaldehyde in 0.1 M phosphate buffer (PB; pH 7.4). The spinal cords were immediately removed and post-fixed in the same fixative agent for 3 h at room temperature. After immersion in 25% sucrose in 0.1 M PB for 48 h at 4 °C for cryoprotection, the lumbosacral spinal cords were quickly frozen using powdered dry ice and then cut into 30 μm-thick horizontal (the lumbar spinal cord: L3–L4 level) and cross- (brain and the lumbosacral spinal cord: L5–S1 level) sections on a cryostat. The endogenous peroxidase activity of the samples was eliminated by incubation in 1% H_2_O_2_ in absolute methanol for 30 min, followed by three 5 min rinsing sessions with phosphate-buffered saline (PBS, pH 7.4). This H_2_O_2_ treatment was omitted for immunofluorescence. After blocking nonspecific binding with 1% normal goat serum and 1% BSA in PBS containing 0.3% Triton X-100 for 30 min at room temperature, the sections were then incubated with the primary rabbit antiserum against GRP (1:2000 dilution) (11081; AssayPro, St. Charles, MO, USA, Research Resource Identifier (RRID): AB_2571636) as described elsewhere [14,29,30,31]. In this study, we performed immunostaining for GRP as the marker for dXL neurons. The specificity of the anti-GRP serum in the spinal cord was performed as demonstrated elsewhere [31]. Immunoreactive products were detected with the Streptavidin-biotin Kit (Nichirei, Tokyo, Japan), followed by diaminobenzidine (Dojindo, Kumamoto, Japan) development according to our method detailed previously [7,11,14,30,31,32]. To determine the density of GRP^+^ fibers in the lumbosacral spinal cord (L5–S1 level), at least ten sections per animal were analyzed using ImageJ software (ImageJ 1.44p, RRID_ SCR_003070) with a set threshold level. The GRP^+^ fiber pixel density was quantified as the average pixel density in the three regions—the SAN and dorsal horn (DH) of each animal, which was calculated as the ratio to the density in the DH in controls in each analysis. The results obtained indicate that the expression of GRP in the spinal DH was involved in the transmission of the itch sensation [31,33]; this distribution of GRP showed no difference with the sex of the subject [7,14].

To examine the activation of GRP^+^ neurons, double immunofluorescence staining for GRP and phosphorylated extracellular signal-related kinases 1 and 2 (pERK), a marker protein for neuronal activation, was performed. After blocking nonspecific binding, as described earlier, the sections were incubated with the anti-pERK (1:1000 dilution) (#9101; rabbit polyclonal antibody, Cell Signaling Technology, Danvers, MA, USA, RRID: AB_331646) [14,34] overnight at 4 °C. After rinsing with PBS, the sections were incubated for 1 h at room temperature with the Dylight 549-linked Fab Fragment Goat Anti-rabbit IgG (1:100 dilution) (Jackson Laboratory, Bar Harbor, ME, USA) [14]. After rinsing, the sections were incubated with 1% H_2_O_2_ in absolute methanol for 20 min at room temperature. After blocking nonspecific binding, the sections were immersed overnight at 4 °C in a 1:200 dilution of GRP antiserum. The second-primary immunoreaction was visualized as green after a 1 h incubation with Alexa Fluor 488-linked anti-rabbit IgG (1:2000 dilution) (Molecular Probes, Eugene, OR, USA, RRID: AB_2576217) [7,9,14].

To determine the distribution of OXT-containing axons, triple immunofluorescence staining was performed for GRP (1:1000 dilution) and OXT-neurophysin (PS60; mouse monoclonal antibody, RRID: CVCL_G254) (1:1000 dilution)—a marker protein for OXT neurons. The PS60 antibody has previously been shown to be specific for OXT neurons at the ultrastructure level [35,36,37]. The Alexa Fluor 555-linked anti-rabbit IgG raised in goats (Molecular Probes, RRID: AB_2535850) [29] and Alexa Fluor 633-linked anti-mouse IgG raised in goats (Molecular Probes, RRID: AB_2535719) were used for detection at a 1:1000 dilution.

### 4.8. Morphological Analysis

We determined the proportion of pERK^+^, GRP^+^, and YFP^+^ neurons in the lumbosacral spinal cord. Immunofluorescence analysis of pERK, GRP, and YFP expression in the neurons of the anterior lumbar spinal cord (L3–L4 level) was performed as described earlier using horizontal sections (approximately 18–22 30 µm-thick sections per animal) [29,30,32]. Briefly, we counted the number of pERK^+^, GRP^+^, and YFP^+^ cell bodies at ×200 magnification in all sections and analyzed a 600 µm^2^ area localized to the midline at the center. Subsequently, we acquired 5–15 micrographs per section, depending on the distribution of the GRP^+^ neurons. These digital micrographs were selected and processed using Adobe Photoshop (Adobe Systems, San Jose, CA, USA) and printed at 300 dots per inch on photographic paper. GRP and YFP neurons were identified by their following characteristics: densely immunostained, anatomical localization (mainly dorsal, dorsolateral, or both to the central canal in the lamina X of the lumbar segments III-IV), relatively large cell bodies (diameters approximately 20–30 µm), and clear round nuclei (diameters approximately 10–15 µm). To avoid overestimating the cell numbers, only pERK^+^ or YFP^+^ and GRP^+^ neurons that contained a round, transected nucleus were counted. As the mean diameter of the nuclei in the GRP neurons was much smaller than the 30 µm-thick sections, this analysis reduced the overestimation of the number of neurons. All micrographs were coded and evaluated without the knowledge of the experimental group designation, and the code was not broken until the analysis was complete.

We next performed a semi-quantitative analysis of the GRP, YFP, and OXT expression. To determine the density of GRP^+^ and YFP^+^ somata and OXT^+^ fibers in the lumbar spinal cord (L3–L4 level), at least 15 horizontal sections (30 µm thick) per animal were captured (magnification, ×42 per section). The optical density of the YFP signals was determined using black-and-white images that were converted from micrographs using ImageJ software (ImageJ 1.44p; National Institutes of Health, Bethesda, MD, USA) according to our established methods [23,30,32]. Briefly, the optical density of the intrinsic fluorescence was estimated by comparison with similar areas of the control sections. The GRP^+^ and YFP^+^ soma pixel density and the OXT^+^ fiber pixel density were semi-quantitated as the total pixel density of each animal, and the data were expressed as the ratio to the density of controls. The results of all immunohistochemical analyses were saved as images and the immunoreactivities were confirmed by multiple researchers.

### 4.9. Statistical Analysis

Statistical analyses were performed using the GraphPad Prism (version 8.4.3; GraphPad Software, San Diego, CA, USA, RRID: SCR_002798). All data are presented as the mean ± SEM and individual dots for each animal. Statistical analyses of the optical density, and the gene expressions between sexually naïve males and sexually experienced males, were assessed using Student’s unpaired *t*-test. The differences in the pERK expression ratio after sexual behavior were performed using a two-way ANOVA. When significant main effects were detected using ANOVA, the post-hoc Tukey’s test was performed. Several analyses in this study were conducted in a “blinded” manner.

## 5. Conclusions

In this study, we revealed the effects of male sexual experience on dXL neurons at the spinal cord level. We found that, in sexually experienced rats, approximately 50% of dXL neurons were activated by both intromission (erection) and ejaculation, whereas, in naïve rats, approximately 30% of dXL neurons were activated by intromission and more than 50% of dXL neurons were activated by ejaculation. We also found that sexual experience increased GRP and OXTR expression at the protein level in dXL neurons. To the best of our knowledge, this is the first demonstration of the effects of sexual experience on molecular expressions in the neural circuits controlling male sexual activity in the spinal cord.

## Figures and Tables

**Table 1:** The proportion of pERK expression in GRP-immunoreactive neurons (%) after sexual behavior.

	Control	Intromission	Ejaculation
Naïve	23.3 ± 3.3	27.3 ± 2.2	56.1 ± 3.2
Experienced	32.5 ± 5.0	45.3 ± 3.5	51.3 ± 3.4

**Table 2:** The proportion of pERK expression in GRP-immunoreactive neurons (%) after reflexive erection or PCA-induced ejaculation.

	Control	Erection	Ejaculation
Reflexive erection	37.8 ± 5.6	42.5 ± 7.8	-
PCA-induced ejaculation	42.1 ± 5.0	-	52.6 ± 3.7

## Data Availability

Not applicable.

## Abbreviations

SEG	spinal ejaculation generator
LSt	lumbar spinothalamic
GRP	gastrin-releasing peptide
dXL	dorsal lamina X of the lumbar spinal cord
SAN	sacral autonomic nucleus
OXT	oxytocin
SNB	spinal nucleus of the bulbocavernosus
OXTR	oxytocin receptor
mPOA	medial preoptic area
IML	intermediolateral cell column
SPFp	subparafascicular thalamic nucleus
pERK	phosphorylated extracellular signal-related kinases 1 and 2
ANOVA	analysis of variance
YFP	yellow fluorescent protein
Tg	transgenic
+	positive
qPCR	quantitative PCR
PCA	*p*-chloroamphetamine
SCI	spinal cord injury
